# Optimism bias and its relation to scenario valence, gender, sociality, and insecure attachment

**DOI:** 10.1038/s41598-022-22031-4

**Published:** 2022-11-02

**Authors:** Mihai Dricu, Dominik A. Moser, Tatjana Aue

**Affiliations:** 1 Institute of Psychology, University of Bern, Bern, Switzerland; 2 Department of Child Psychiatry, Cantonal University Hospital of Vaud, Lausanne, Switzerland

**Keywords:** Human behaviour, Social behaviour, Risk factors

## Abstract

Optimism bias refers to the tendency to display unjustified high/low expectations of future positive/negative events. This study asked 202 participants to estimate the likelihood of 96 different events. We investigated optimism biases for both oneself and the general population, and how these biases are influenced by gender, valence of the event, sociality of the event, as well as attachment anxiety and attachment avoidance. We found that sociality interacted with gender, with the difference in optimism bias for social vs. alone events being larger among women than among men. Attachment anxiety mainly reduced the optimism bias among men deliberating over future alone situations, while attachment avoidance primarily reduced optimism bias among female respondents deliberating over future social interactions. These results may have implications for the well-being and motivation of differently attached men and women and ultimately inspire psychotherapy interventions.

## Introduction

Optimism bias refers to the tendency to display unjustified high expectations of future positive outcomes and/or unjustified low expectations of future negative events^1–3^. Research has shown that most people are in fact overly optimistic about their personal future^4,5^, but one pending question is whether this optimism bias results from expecting more positive outcomes or less negative ones than would otherwise be warranted^6–9^. Most previous studies have predominantly focused on negative outcomes such as accidents and illnesses. In addition, the majority of studies comparing optimism for both positive and negative outcomes have not matched these outcomes on critical characteristics such as perceived frequency or controllability in the general population^6^, which can significantly influence the magnitude of optimism displayed^5,10^. Hence, to adequately investigate valence aspects in optimism bias, the current work controlled for influences of diverse critical characteristics.

Another open question concerns gender differences in optimistic expectancies. Some studies found that men are more optimistic than women, but these findings relate to financial and economic contexts (e.g.^11–13^), where men and women have different perceptions of and attitudes toward risk (e.g., on average, more males than females favor risk^12,14–16^). Other studies focused on a single positive event (“having a happy marriage”) and a single negative event (“getting divorced”), which were not matched on any characteristics apart from their complementarity^17,18^. These studies also revealed that men were more optimistically biased than were women—but only for the negative event. Yet, to draw firm conclusions about gender differences in optimism bias, a broader consideration of multiple positive vs. negative scenarios is strongly indicated. To our knowledge, no studies have investigated gender differences in optimistic expectations across a diverse range of both positive and negative situations.

Our investigation hence aimed to fill these two gaps in knowledge—valence aspects and gender differences in optimism bias—by asking male and female respondents to rate for themselves and for the general population the chances of experiencing a series of positive and negative events. These events had been validated and selected so that they are matched on five key event characteristics, namely perceived frequency in the general population, controllability, valence (i.e., deviation from neutral), and emotional intensity. Correspondingly, our construction of the events allowed for the determination of how respondents’ answers for themselves deviate from their own expectations for the general population: Do they display lowered expectancies for the negative events (for themselves compared to the general population); do they reveal heightened expectancies for the positive events; or are they characterized by both?

Notably, the current investigation had a third and final aim, namely whether the sociality of future outcomes moderates the magnitude of the optimism bias. There is consensus that people evaluate themselves differently from other people and they also evaluate social interactions differently from single-person scenarios^19–21^. Specifically, social interactions represent a meeting of two minds. Thus, when estimating future happenings, the individual not only takes the self into account but also other people. Moreover, because one has limited control over other people’s comprehension, reactions, and opinions, deliberating over social interactions (compared with reflecting on one’s individual actions) is inherently more complex. It remains, however, unanswered whether such varying complexity has an impact on the expected likelihood of future events. For example, do people show similar overoptimism when they anticipate enjoying a quiet afternoon by oneself and enjoying a vacation together with the significant other? How about having an argument with a relative and having a migraine? To answer this question, our positive and negative events contained an equal number of alone and social situations. To our knowledge, to date, no other study has taken the sociality of future outcomes into consideration when investigating optimism bias.

We tested the first two research questions (valence question and gender question) separately from the third one (sociality question; see Table 1). To assess gender differences in optimism bias and to determine whether the optimism bias arises because people display lowered expectancies for personal negative events or heightened expectancies for personal positive ones (valence question), we looked at the variables *target* (self vs. general population), *valence* (positive vs. negative situations), and *gender* (male vs. female). We expected that the respondents would rate positive and negative events in the general population as equally likely (in accordance to how we validated the target events) but that, overall, they would expect more positive than negative events when thinking about their own future (H1: interaction between target and valence of events on likelihood estimates). We predicted that this optimism bias would be generated by both participants displaying lowered estimates for personal negative events and enhanced estimates for positive personal events (compared to the general population estimations). Lastly, we expected that men would display a stronger optimism bias than would women and that this effect would be driven mainly by men more strongly downsizing their personal likelihood of facing negative events (compared to the one specified for the general population) than women^11–13^ (H2: interaction between target, valence of events, and gender on likelihood estimates).

**Table 1:** List of hypotheses for personal optimism.

ID	Effect	Predicted direction
H1	Target × valence^+^	H1: Desirable − Undesirable: Self > General Population
		H1a: General Population: Desirable = Undesirable^+^
		H1b: Self: Desirable > Undesirable^+^
H2	Target × valence × gender^−^	H2: Desirable − Undesirable (resulting from responses for the Self): Male > Female^−^
		H2a: General population, Male: Desirable = Undesirable^+^
		H2b: General population, Female: Desirable = Undesirable^+^
		H2c: Self, Male: Desirable > Undesirable^+^
		H2d: Self, Female: Desirable > Undesirable^+^
H3	Sociality^−^	Self: Social > Alone^−^
H4	Sociality × gender^+^	Female > Male: Social-Alone^+^
H5	Sociality × valence^−^	Self: (Desirable > Undesirable)_social_ > (Desirable > Undesirable)_alone_^−^
H6	Sociality × valence × gender^−^	Female > male: (Desirable − Undesirable)_social_ − (Desirable − Undesirable)_alone_^−^
H7	Sociality × attachment avoidance^−^	Self, highly avoidant: Alone > Social^−^
H8	Sociality × attachment avoidance × valence^+^	Self, highly avoidant: (Desirable > Undesirable)_alone_ > (Desirable > Undesirable)_social_^+^
H9	Sociality × attachment anxiety^−^	Self, highly anxious: Social > alone^−^
H10	Sociality × attachment anxiety × valence^+^	Self, highly anxious: (Desirable > Undesirable)_social_ > (desirable > undesirable)_alone_^+^

The dependent variable was the likelihood estimate (ranging from 0 to 100%). The superscripts refer to hypotheses that were supported (^+^) and not supported (^−^) by the analyses.

To assess the effect of sociality on optimism bias (i.e., sociality question), we looked at the estimations that the respondents made for themselves (ignored the general population) and kept the variables *valence* and *gender* while adding *sociality* (two levels: alone and social events). Because of a lack of direct precedent in the optimism literature which, to date, has ignored sociality aspects, we based our hypotheses about the sociality of future outcomes on attachment theory. Attachment theory posits that the habitual interactions with our caregivers during childhood continue into adulthood as cognitive and emotional schemas^22,23^ that influence how adults perceive themselves and others^21,24^. We therefore included *attachment anxiety* (ANX) and *attachment avoidance* (AVO) as additional continuous predictors of optimism bias. Notably, it appears important to investigate attachment (and sociality) together with gender and in the context of event valence, as the literature on the topic indicates their interrelatedness^25–27^. It is necessary to consider the multiple interactions between those interrelated factors in the current study, because focusing on them individually and subsequently could easily lead to wrong intermittent conclusions.

Because we all strive to achieve meaningful and mutually caring relationships with others^22,28^, human interactions are at the forefront of our minds (on account of an availability heuristic^29^). As a result, we expected that respondents would generally rate social situations as more frequent than alone situations (H3: main effect of sociality of scenarios on likelihood estimates), and that this effect would be more pronounced for women than for men (H4: interaction effect between sociality of scenarios and gender on likelihood estimates; see research on stronger preference of women than men for social stimuli, e.g.^30,31^). Moreover, we hypothesized that, because of higher personal relevance, respondents would manifest stronger optimism for social events than alone events (H5: interaction between sociality and valence of events on likelihood estimates) and that this difference would be more pronounced for women than for men (H6: interaction between gender, sociality, and valence of events on likelihood estimates).

Although optimism bias is believed to be a basic human heuristic^32^, with advantages for health and subjective well-being^33,34^, attachment styles in the form of cognitive and emotional schemas about self and others^21,23,24^ should moderate the effects of the *valence* of events on the likelihood estimates (i.e., the magnitude of optimism bias) depending on the *sociality* of events. *AVO* predisposes individuals toward chronic self-reliance, meaning more time engaged in alone situations than social situations^35–38^. This is fueled by a positive image of oneself and a negative image of others^24,39^. By contrast, *ANX* predisposes individuals toward clinging and co-dependent behaviors, as well as gravitation toward social interactions and away from alone situations^36,38,40^. This is fueled by a negative image of oneself and a positive image of others^24,39^. Considering this research, we anticipated that respondents high on *AVO* would rate social situations to be overall less frequent than alone situations (H7: interaction between sociality and attachment avoidance on likelihood estimates), and that optimism bias would be smaller in social vs. alone scenarios (H8: interaction between sociality, valence, and attachment avoidance on likelihood estimates). By contrast, we predicted that respondents high on *ANX* would expect alone situations to be overall less likely to occur than social situations (H9: interaction between sociality and attachment anxiety on likelihood estimates), and that their optimism bias would be smaller for alone vs. social situations (H10: interaction between sociality, valence, and attachment avoidance on likelihood estimates). Lastly, we made no predictions about gender differences in (a) attachment styles or (b) the moderating effects of attachment styles on the effects of sociality and valence, because previous research did not reveal consistent gender differences in *ANX* and *AVO*^41–43^.

## Results

### Descriptive statistics

Two independent t-tests (two-tailed) were performed on scores of *ANX* and *AVO*, respectively to determine potential gender differences. Male respondents (M = 2.89, SD = 0.75) reported lower *ANX* scores than did female respondents (M = 3.27, SD = 0.78; t (200) = 3.35, p < 0.001, Cohen’s d = 0.497). Similarly, male respondents (M = 2.95, SD = 0.52) reported lower *AVO* scores than did female respondents (M = 3.06, SD = 0.58; t (200) = 1.88, p < 0.001, Cohen’s d = 280). Both dimensions of *ANX* (Shapiro–Wilk normality test W = 0.988, p = 0.091) and *AVO* (Shapiro–Wilk normality test W = 0.988, p = 0.074) had a normal distribution of scores. Levene’s test showed that the variances for *ANX* (F (1,200) = 0.703, p = 0.403) and *AVO* (F (1,200) = 0.783, p = 0.377) were equal for male and female respondents. Finally, scores of *ANX* and *AVO* were equally positively correlated among female respondents (Pearson’s r (133) = 0.407, p < 0.001) and male respondents (Pearson’s r (69) = 0.302, p = 0.012; comparison male–female: z = 0.80, p = 0.42).

### Hypotheses H1, H2 (focusing on the valence and gender questions)

**Figure 1:**
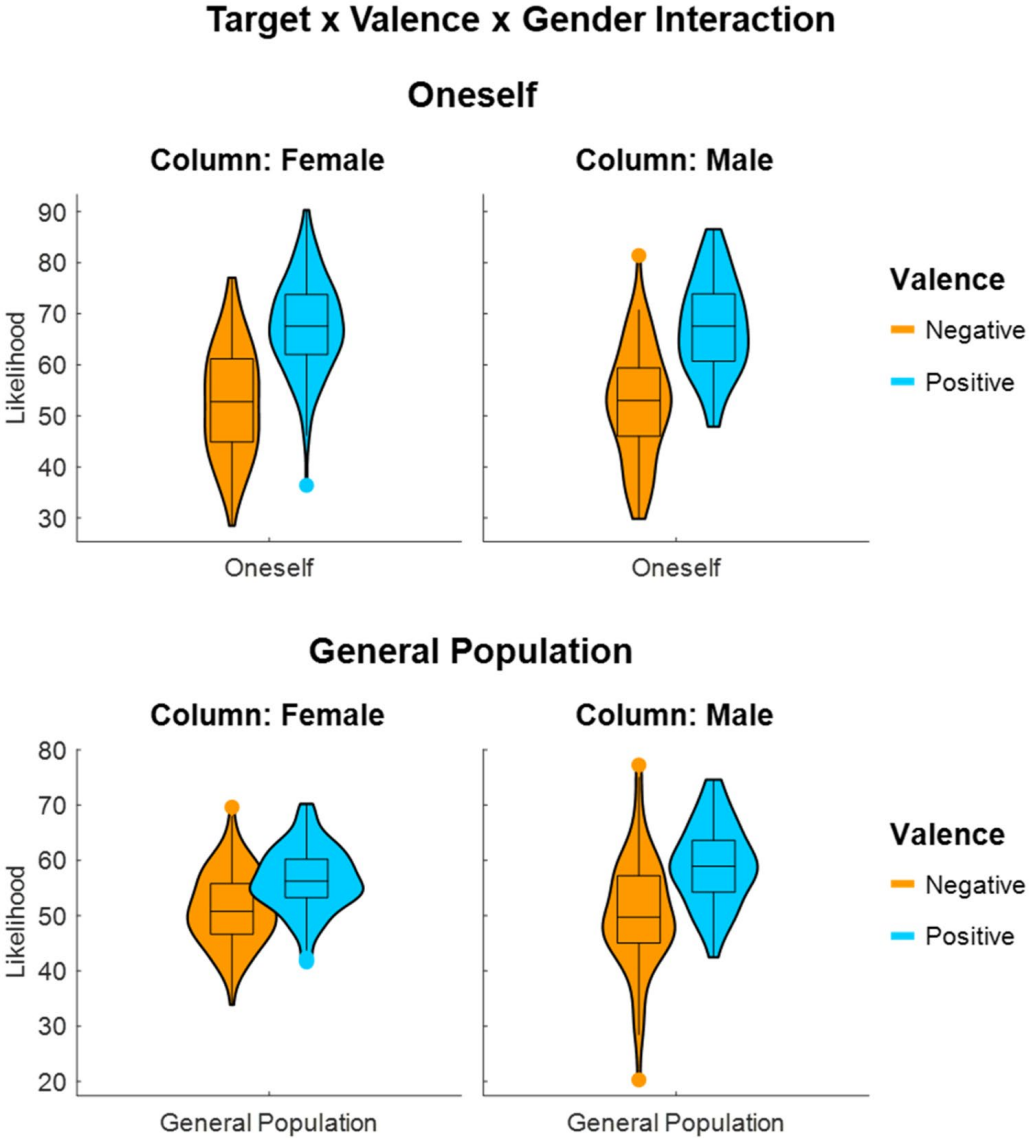
Violin plots displaying the interaction between target, valence, and gender.

The first linear mixed model (using self and general population as targets, positive and negative valence, and male and female gender; complete results in Supplementary Table S1) yielded a significant interaction effect between *target* and *valence* (H1: F (1,19,138.0) = 147.87, p < 0.001), revealing, as expected, that respondents rated positive and negative events as equally likely to occur in the general population (H1a: t (46.5) = 1.54, p = 0.131; M_pos_ = 57.8%, M_neg_ = 50.8%), whereas they rated themselves as more likely to encounter the positive situations than the negative ones (H1b: t (46.5) = 3.32, p = 0.002; M_pos_ = 67.5%, M_neg_ = 52.5%). Additionally, there was a significant three-way interaction effect between *target*, *valence,* and *gender* (H2: F (1,19,138.0) = 7.03, p = 0.008; Fig. 1), that we unpacked first by target and then by gender. Specifically, the positive and the negative outcomes were rated with similar likelihood in the general population by both male (H2a: M_diff_ = 8.76%, t (48.2) = 1.92, p = 0.061; M_pos_ = 59.1%, M_neg_ = 50.3%) and female (H2b: M_diff_ = 5.17%, t (46.9) = 1.14, p = 0.261; M_pos_ = 56.5%, M_neg_ = 51.4%) respondents. However, an optimism bias (i.e. the difference between estimates for positive events and negative events) was displayed by both male (H2c: M_diff_ = 15.07%, t (48.2) = 3.30, p = 0.002; M_pos_ = 67.4%, M_neg_ = 52.3%) and female (H2d: M_diff_ = 15.00%, t (46.9) = 3.31, p = 0.002; M_pos_ = 67.7%, M_neg_ = 52.7%) respondents when they evaluated their own likelihood of experiencing the events (non-significant interaction valence × gender for evaluating oneself: F (1, 9446) = 9.48, p = 0.004). Post hoc independent group t tests based on aggregated data indicated that the optimism bias (positive–negative events) for self vs. general population was larger for women than men (t (200) = 2.20, p = 0.029), going in opposite direction of H2.

This optimism bias when evaluating oneself was driven exclusively by heightened expectancies regarding the chances of experiencing positive events compared to the general population and this pattern was present for both female (M_diff_ = 11.16%, t (19,138) = 20.34, p < 0.001) and male (M_diff_ = 8.32%, t (19,138) = 10.92, p < 0.001) participants. Both men (M_diff_ = 2.0%, t (19,138) = 2.63, p = 0.009) and women (M_diff_ = 1.33%, t (19,138) = 2.42, p = 0.016) were also characterized by heightened expectancies of experiencing negative events (compared with the general population). However, these differences were overcompensated by the effect observed for positive events (i.e., differences between self and general population were stronger for positive than negative events), so that the net result was a significant optimism bias present for both genders (see above for H2c and H2d).

Furthermore, there were a main effect of *valence* (F (46.1) = 5.93, p = 0.019; M_pos_ = 62.7%, M_neg_ = 51.7%) and an interaction effect between *valence* and *gender* (F (19,138.0) = 7.59, p = 0.006; males: M_pos_ = 63.3%, M_neg_ = 51.3%; females: M_pos_ = 62.1%, M_neg_ = 52.0%), that were both driven exclusively by the ratings for the self, as seen in the interactions above. Lastly, there was no main effect of *gender* (F (200) = 0.07, p = 0.796) nor an interaction between *target* and *gender* (F (19,138.0) = 2.66, p = 0.103).

### Hypotheses H3-H10 (focusing on the sociality question)

**Figure 2:**
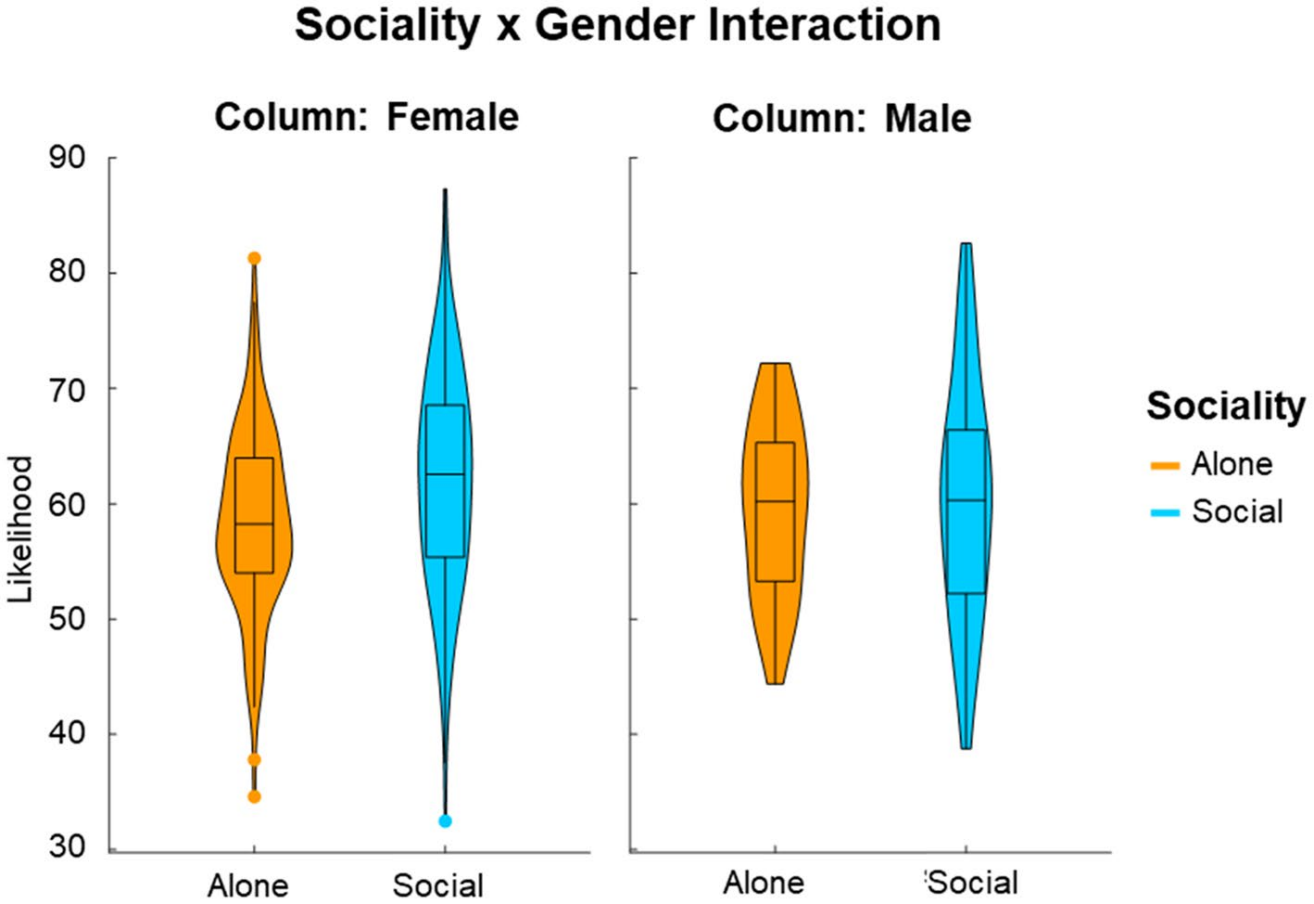
Violin plots displaying the interaction between sociality and gender.

The second linear mixed model (complete results in Supplementary Table S2) did not reveal the predicted significant main effect of *sociality* (H3; F (1,44.2) = 0.170, p = 0.682), suggesting that the alone (M = 59.0%) and the social events (M = 61.0%) did not differ concerning the respective overall likelihood participants assigned to them. The predicted interaction effect between *sociality* and *gender* was significant (H4; F (1,9432) = 7.100, p = 0.008; Fig. 2). The simple effects revealed that the male and female respondents each estimated the likelihood of alone and social events to be similar (male: t (46.3) = 0.09, p = 0.923; female: t (44.6) = 0.73, p = 0.472) and that there was no significant gender difference for either alone or social scenarios (alone: t (286) = 1.15, p = 0.251; social: t (286) = − 1.07, p = 0.288). Yet, post hoc tests using aggregated data revealed (consistent with our H4) that the difference between the likelihood ratings for social vs. alone events was more substantial among women than among men (t (200) = − 2.59, p = 0.01), as women gave nominally lower likelihood estimates than men during the alone condition (women: M = 58.3%, men: M = 59.8%, t (286.3) = 1.15, p = 0.251) and nominally higher likelihood estimates during the social condition (women: M = 61.7%, men: M = 60.3%; t (286.3) = 1.06, p = 0.288). The hypothesized significant interaction effects between *sociality* and *valence* (H5: stronger optimism bias in social than alone scenarios; F (1, 44.2) = 0.017, p = 0.897), or between *sociality*, *valence,* and *gender* (H6: stronger optimism bias in social than alone scenarios in females compared with males; F (1,9432) = 0.403, p = 0.526) were not significant.

**Figure 3:**
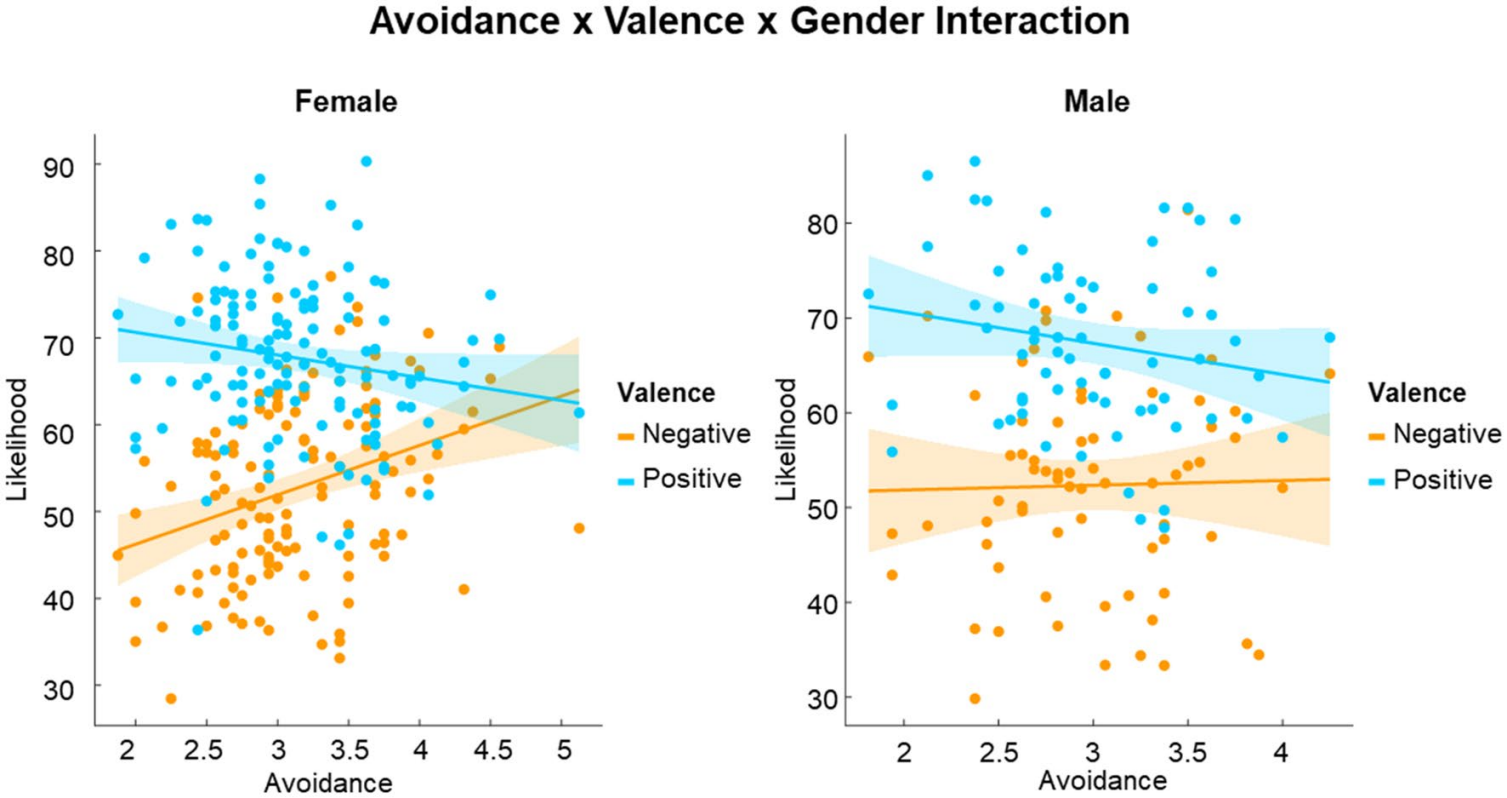
Scatter plots displaying the interaction between attachment avoidance, valence, and gender.

The predicted interaction effect between *sociality* and *AVO* (H7; F (1,9432) = 0.526, p = 0.469) was not significant either, suggesting that variations in *AVO* did not affect the overall differentiation of social vs. alone scenarios. However, *AVO* moderated the magnitude of optimism bias (interaction *AVO* × *valence*: F (1,9432) = 12.07, p < 0.001), and this was further dependent on the *gender* of the participants (interaction *AVO* × *valence* × *gender*; F (1,9432) = 7.233, p = 0.007; Fig. 3) and the s*ociality* of the outcomes (H8: interaction *AVO* × sociality × *valence*; F (1,9432) = 4.852, p = 0.028). The simple effects revealed that optimism bias was present in all respondents, regardless of their levels of *AVO*, but that its magnitude decreased as scores of reported *AVO* increased (Mean *AVO* – 1SD: M_diff_ = 16.60%, t (45.5) = 3.52, p < 0.001; Mean *AVO*: M_diff_ = 14.5%, t (44.2) = 3.10, p = 0.003; Mean *AVO* + 1SD: M_diff_ = 12.5%, t (45.8) = 2.64, p = 0.011). This decrease was sharper among female respondents (Mean *AVO *− 1SD: M_diff_ = 19.40%, t (46.4) = 4.10, p < 0.001; Mean *AVO*: M_diff_ = 15.8%, t (44.6) = 3.36, p = 0.002; Mean *AVO* + 1SD: M_diff_ = 12.1%, t (46.2) = 2.56, p = 0.014) than male respondents (Mean *AVO *− 1SD: M_diff_ = 13.70%, t (49.7) = 2.85, p = 0.006; Mean *AVO*: M_diff_ = 13.3%, t (46.3) = 2.80, p = 0.007; Mean *AVO* + 1SD: M_diff_ = 12.8%, t (51.2) = 2.64, p = 0.011), and also sharper for social outcomes (Mean *AVO *− 1SD: M_diff_ = 18.5%, t (45.5) = 2.77, p = 0.008; Mean *AVO*: M_diff_ = 15.1%, t (44.2) = 2.29, p = 0.027; Mean *AVO* + 1SD: M_diff_ = 11.8%, t (45.8) = 1.76, p = 0.084) than for alone outcomes (Mean *AVO *− 1SD: M_diff_ = 14.7%, t (45.5) = 2.20, p = 0.033; Mean *AVO*: M_diff_ = 13.9%, t (44.2) = 2.10, p = 0.041; Mean *AVO* + 1SD: M_diff_ = 13.2%, t (45.8) = 1.97, p = 0.055). The gender of respondents and the sociality of events did not have multiplicative effects on the moderation of *AVO* on optimism bias, as suggested by the lack of a significant four-way interaction *AVO* × sociality × *valence* × *gender* (F (1.9432) = 0.792, p = 0.373). Although at high levels of *AVO* (Mean + 1SD), there was no significant difference in the assessment of alone and social outcomes (F (1,57.5) = 1.09, p = 0.300), the pattern of a sharper decrease in optimism bias for social outcomes than alone outcomes is consistent with our hypothesis H8.

**Figure 4:**
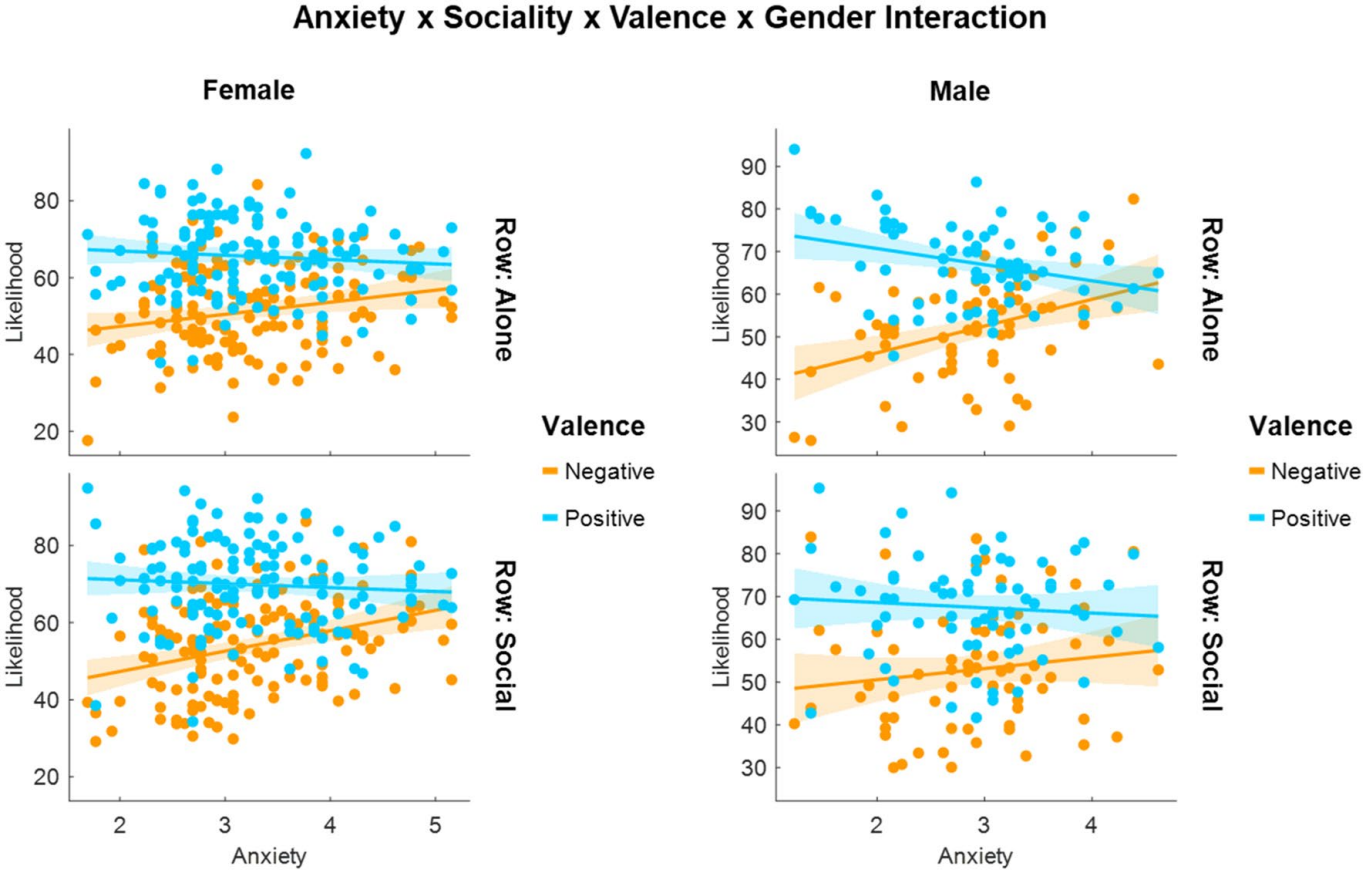
Scatter plots displaying the interaction between attachment anxiety, sociality, valence, and gender.

Similarly, the predicted interaction effect between *sociality* and *ANX* (H9; F (1,9432) = 0.487, p = 0.485) was not significant, suggesting that *ANX* did not affect the overall differentiation of social vs. alone scenarios. However, *ANX* moderated the magnitude of optimism bias (interaction *ANX* × *valence*: F (1,9432) = 45.743, p < 0.001), and this was further dependent on the *sociality* of the outcomes (H10: interaction *ANX* × *valence* × *sociality*; F (1,9432) = 5.110, p = 0.02431), the *gender* of the participants (interaction *ANX* × *valence* × *gender*; F (1,9432) = 5.415, p = 0.020; Supplementary Fig. S1), and both the *gender* and the *sociality* (interaction *ANX* × *valence* × *sociality* × *gender*; F (1,9432) = 5.288, p = 0.021; Fig. 4). Although optimism bias was present in all respondents, regardless of the levels of *ANX*, its magnitude decreased as scores of reported ANX increased (Mean *ANX *− 1SD: M_diff_ = 18.50%, t (45.3) = 3.93, p < 0.001; Mean *ANX*: M_diff_ = 14.5%, t (44.2) = 3.10, p = 0.003; Mean *ANX* + 1SD: M_diff_ = 10.6%, t (46.0) = 2.23, p = 0.030). This decrease was sharper among male respondents (Mean *ANX *− 1SD: M_diff_ = 18.61%, t (48.1) = 3.89, p < 0.001; Mean *ANX*: M_diff_ = 13.28%, t (46.3) = 2.80, p = 0.007; Mean *ANX* + 1SD: M_diff_ = 7.95%, t (52.3) = 1.63, p = 0.109) than female respondents (Mean *ANX *− 1SD: M_diff_ = 18.39%, t (47.0) = 3.87, p < 0.001; Mean *ANX*: M_diff_ = 15.78%, t (44.6) = 3.36, p = 0.002; Mean *ANX* + 1SD: M_diff_ = 13.18%, t (46.0) = 2.79, p = 0.008), and also sharper for alone outcomes (Mean *ANX *− 1SD: M_diff_ = 19.2%, t (45.3) = 2.89, p = 0.006; Mean *ANX*: M_diff_ = 13.92%, t (44.2) = 2.10, p = 0.041; Mean *ANX* + 1SD: M_diff_ = 8.63%, t (46.0) = 1.29, p = 0.203) than social outcomes (Mean *ANX *− 1SD: M_diff_ = 17.78%, t (45.3) = 2.67, p = 0.010; Mean *ANX*: M_diff_ = 15.14%, t (44.2) = 2.29, p = 0.027; Mean *ANX* + 1SD: M_diff_ = 12.50%, t (46.0) = 1.87, p = 0.068). Additionally, sociality and gender had a multiplicative effect on the moderating effect of *ANX* on optimism bias. Specifically, optimism bias decreased as a function of increasing scores of *ANX* and this decrease was the sharpest for male respondents in alone situations (Mean *ANX *− 1SD: M_diff_ = 23.08%, t (47.0) = 3.15, p = 0.003; Mean *ANX*: M_diff_ = 15.53%, t (44.0) = 2.15, p = 0.023; Mean ANX + 1SD: M_diff_ = 7.98%, t (47.0) = 1.09, p = 0.282). Although at high levels of *ANX* (Mean + 1SD), there were no significant differences in the assessment of alone and social outcomes (F (1,52.8) = 0.19, p = 0.664), the pattern of a sharper decrease in optimism bias for alone outcomes than social outcomes is consistent with our hypothesis H10.

## Discussion

Our study had three aims: (1) To reveal whether optimism bias stems from heightened expectations of self-relevant positive future outcomes and/or lowered expectations of self-relevant negative future outcomes (valence question), (2) to determine whether there are gender differences in the magnitude of optimism (gender question), and (3) whether the sociality of future outcomes (in combination with personal attachment tendencies) plays a role in optimism bias (sociality question).

Our first aim has not been entirely satisfactorily addressed by the previous literature in the field. For example, some studies chose negative events for which epidemiological data or national statistics exist (e.g., “having cancer”, “getting divorced”) and constructed positive events as their complement (“not having cancer”, “not getting divorced”^17,18,44^). Other studies used two unique sets of negative and positive events, but they did not match them on potentially confounding characteristics such as controllability or frequency^45–48^. A more in-depth comparison of optimism bias toward positive vs. negative outcomes should be based on events that are matched on as many potentially confounding characteristics as possible^5,10^. In our study, therefore, we chose a balanced set of positive and negative events that had been matched on multiple characteristics: perceived frequency in the general population, controllability, emotional intensity, and valence (i.e., how strongly an event’s valence deviates from a hypothetical midpoint, implying an emotionally neutral event). We then asked participants to rate their own likelihood as well as that of the general population to experience a series of positive and negative events.

Respondents expected the positive and negative events to occur with equal chances in the general population, but they expected significantly more positive than negative events for themselves, yielding support to our hypothesis H1. Participants displayed heightened expectancies regarding both positive and negative events for themselves compared to the general population, strengthening the account of egocentric biases that make people think about themselves more readily, more effortlessly and more vividly than about other individuals^46,49,50^. However, the effect for positive outcomes was substantially higher than the one observed for negative outcomes, and a net optimism bias arose. As such, our study shows that optimism bias is driven by heightened expectations of self-relevant positive future outcomes. This finding is in line with motivational accounts of optimism bias: People wish to experience positive outcomes, and their deliberation process may reflect these desires^7,51–54^. Furthermore, because thinking about positive outcomes is in itself enjoyable it can incentivize individuals to engage more in it and less in the consideration of negative outcomes. This can hence result in a superficial evaluation of negative outcomes compared to the positive ones, which might ultimately lead to the optimism bias observed^7^.

For our second research question, we explored potential gender differences in the magnitude of optimism bias. Previously, studies have suggested that men might be more optimistic than women^11–13,17,18^. Our study found no support for globally stronger magnitudes of optimism bias in men than women. While both men and women expected more positive than negative outcomes for their personal future, somewhat surprisingly, the effect observed was stronger in women than men (countering our H2; also, we found no support for our H6). Moreover, our study suggests that direction and strength of gender differences are shaped by the nature of the events investigated as well as attachment characteristics. This was true for both model 1, where gender interacted with the target (self vs. general population) and valence (H2), as well as for model 2, where there were also numerous three- and four-way interactions involving gender with sociality (alone vs. social), valence, and attachment measures (see next paragraph for specifications). Our study therefore suggests that it very much matters how studies assess optimism bias and what other factors are included when it comes to gender differences.

Third, we wanted to determine whether the magnitude of optimism bias is moderated by the sociality of the target event. For example, do people equally deliberate the chance of having food poisoning and the chance of having an argument with a relative? Our results suggest that respondents overall manifest a similarly sized optimism bias regardless of whether the future event concerns the respondent alone or an interaction with someone else. However, attachment styles moderated this relationship. Increasing scores of attachment insecurity (i.e., either high avoidance or high anxiety) decreased the magnitude of optimism bias both independently from the gender of respondents and the sociality of the outcomes as well as synergistically with them. High scores on attachment avoidance and attachment anxiety acted in complementary patterns and in line with our hypotheses (H8, H10). Moreover, this was further stratified by gender effects. Specifically, high avoidance decreased the magnitude of optimism bias significantly more for social than alone outcomes and more among female than male respondents. By contrast, high scores of attachment anxiety decreased optimism bias more strongly for alone than social outcomes and more among male than female respondents. Interestingly, in contrast to attachment avoidance, attachment anxiety also had multiplicative effects with the gender of the participants and the sociality of the outcomes: of all our gender-sociality combinations, high scores of attachment anxiety decreased optimism bias the most among male respondents in alone outcomes.

The effects of the sociality of outcomes and attachment insecurity on optimism bias are in line with our hypotheses and with the past literature. Individuals with an avoidant attachment style are predisposed toward chronic self-reliance and engage more in alone versus social situations^35–38^, on account of a positive image of themselves and a negative image of others^24,39^. Such a working model of self-others is expected to eliminate an otherwise normal optimism bias for social interactions. By contrast, anxiously attached individuals exhibit clinging and co-dependent behaviors and they gravitate toward social interactions and away from alone situations^36,38,40^, on account of a negative image of themselves and a positive image of others^24,39^. This predisposes anxious individuals to a lack of optimism bias for alone situations.

Interestingly, attachment insecurity interacted with the gender of respondents to moderate optimism bias in our study. We are not aware of other studies reporting interactions between gender and attachment dimensions to predict any psychological construct. Our study suggests that anxious males and avoidant females are particularly at risk for an absent optimism bias (otherwise a marker of mental health^6^), which, in turn, is predictive of lower subjective well-being^34,55^ and is a risk factor for depression^56^. Future studies in positive and clinical psychology should remain aware of these gender-attachment insecurity interactions, which may affect some of the findings. Specifically, it might be worthwhile to identify potential attachment insecurities in order to develop individually tailored interventions that focus on strengthening resources in the most critical conditions. Whereas it may be indicated to target optimism in alone situations for male patients with high attachment anxiety, treatment of women with high attachment avoidance may best benefit from a consideration of optimistic expectancies in social situations. Before drawing firm conclusions on these points, however, our results clearly need to be replicated and further explored in subsequent studies.

Our research may be criticized on several accounts. First, our participant sample of young (18–30 years old) full time-students cannot be considered as representing the general population. The current findings hence need to interpreted against this backdrop and replicated with a more diverse (in terms of age, social and financial background, gender, culture, and ethnicity) sample. Second, we acknowledge that there might be several interesting effects that our research was unable to uncover. To keep statistical complexity and interpretability of the data at an acceptable level, we decided against including the variable *target*, when investigating our hypotheses H3–H10. Therefore, the current research clearly does not negate the existence of interesting interactions between *target* and *sociality*, *ANX*, and *AVO* (and their potential interplay with *valence* and *gender*).

In sum, our study sheds light on the complexity of human social behavior and its associated biases and shows that men and women can be affected differently depending on their kind of attachment insecurity and the nature of the situation under investigation.

## Methods

### Experimental design

The design was mixed, with three within-subject factors pertaining to the scenarios (*valence*: positive and negative; *sociality*: alone and social; *target*: self and general population) and one between-subject factor (*gender*: male and female). Additionally, *attachment anxiety (ANX)* and *attachment avoidance (AVO)*, measured with the short version of the Attachment Style Questionnaire (ASQ^57^), were used as continuous predictors. The ASQ-short form consists of 29 items (16 items measuring AVO and 13 items measuring ANX). Responses are given on a 6-point scale (“totally disagree” to “totally agree”). The questionnaire’s two factor structure has been investigated in relation to other attachment questionnaires and successfully validated. Reported internal reliability and retest reliability both amount to α > 0.86^58^. In the current study, Cronbach’s α for the attachment anxiety subscale was 0.87, and the one for the attachment avoidance subscale was 0.78.

### Stimuli (alone-social optimism bias scenarios; ALSO-OBS)

Our stimuli consisted of 48 scenarios (12 positive-alone, 12 positive-social, 12 negative-alone, 12 negative-social) that were balanced on (a) extent of deviation from theoretical midpoint in the direction of alone vs. social, (b) valence deviation from neutral, (c) frequency in the general population, (d) controllability, and (e) emotional intensity. To achieve this balancing, an initial pilot study was run. In a first step, we brainstormed social and alone events. A starting place were the 32 events used in^59^. Those events had not been generated with the alone vs. social attribute in mind and were incidentally skewed towards social-positive and alone-negative situations. Therefore, the current authors brainstormed as many scenarios as possible per each of the 2 × 2 design quadrants (social vs. alone; positive vs. negative), placing particular emphasis on the underrepresented quadrants (i.e., social-negative and alone-positive outcomes). These scenarios were brainstormed with two constraints in mind. First, they must reflect everyday situations that most people encounter without ‘favoring’ any particular social group. This was paramount to ensure that all participants could rightfully imagine themselves in those situations. Second, the situations had to reflect both rare and frequent events closer to the mean rather than extremely rare or extremely frequent situations, because the latter have raised concerns that they might artificially increase the magnitude of the optimism bias^46^.

Following these efforts and constraints, we generated one hundred and seventy-seven events, including the original events in Dricu et al.^6^. We then collected data on five perceived event characteristics (i.e., perceived frequency in the general population, controllability, (deviation from neutral) valence, emotional intensity, and personal experience with the event under investigation) among one hundred and nineteen respondents from Germany (n = 95), Austria (n = 20) and Switzerland (n = 5), who were recruited on www.prolific.co (age range 18–49 years old; M = 27.6 years old; SD = 6.9; 72 males and 47 females). Next, we determined the average scores for each of the five event characteristics separately for each of the one hundred and seventy-seven events. We removed fifty-seven events whose 95% confidence interval included the value “50” (i.e., the lower CI value was smaller than 50% and the higher CI value was larger than 50%) on any scale, ending up with 120 events. Subsequently, we agreed on the final pool of 48 scenarios by using a jackknife technique. This technique is a simple and popular resampling method that allows estimation of bias and variance in a sample and can then appropriately reduce the bias within the sample^60^. We added events in the analysis while excluding others until the events were perfectly balanced on the five event characteristics. The complete battery of stimuli used is presented in the Supplementary Table S5. Following their target evaluation (likelihood estimate) in the current study, we also asked participants what their perceived valence (from − 50 to 50) and sociality (from 0 to 100) of each scenario was (manipulation check).

**Figure 5:**
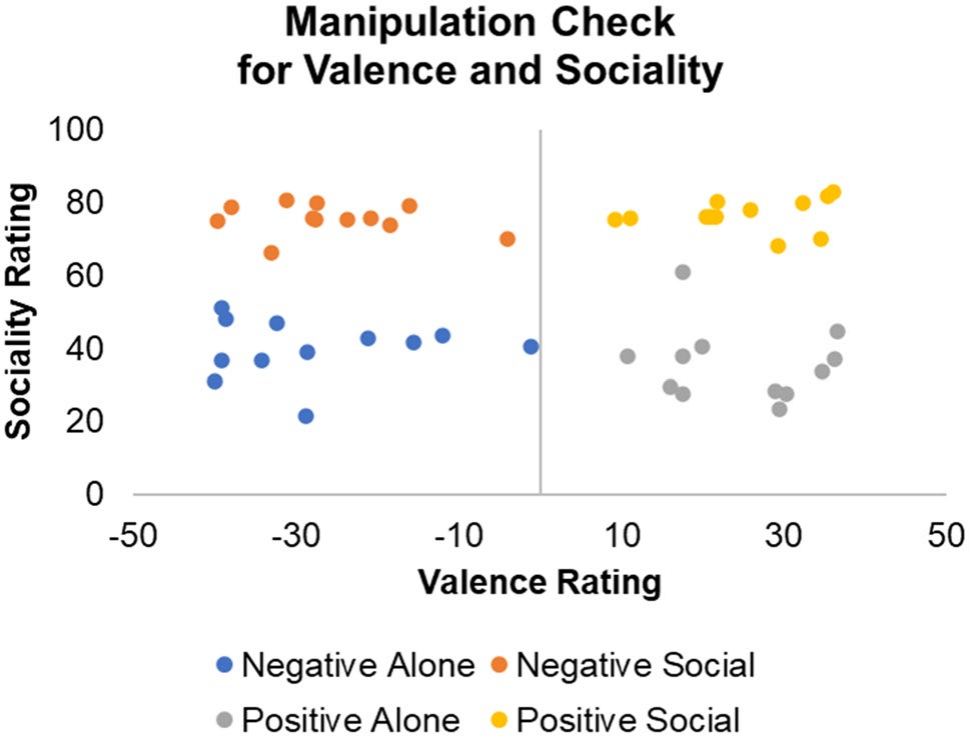
Manipulation check for valence and sociality of the selected scenarios.

Subsequent to data acquisition, we performed manipulation checks for all 48 events using a 2 (perceived valence: negative vs. positive) × 2 (perceived sociality: alone vs. social) design. We found that all events were rated as expected: Negatively valenced events all had a negative mean valence, and positively valenced events all had a positive mean valence, while perceived sociality was consistently higher for social scenarios than for alone scenarios. Importantly, there was no overlap between the stimuli belonging to the resulting four different categories (M [SD] of the different categories: (a) social-negative: valence = − 25.7 [9.9], sociality = 75.6 [4.2]; (b) alone-negative: valence = − 27.6 [12.6], sociality = 39.9 [8.1]); (c) social-positive: valence = 24.9 [9.0], sociality = 76.7 [4.4]; (d) alone-positive: valence = 24.6 [9.0], sociality = 35.7 [10.2]; see Fig. 5).

### Participants

Although there is no gold standard for determining a minimum sample size when designing linear mixed models^61,62^, we chose a sample size of 200 participants, which is double than the most conservative recommendation^63^. For our study, participants were recruited via the Department of Psychology’s SONA system of a Swiss university (for reasons of convenience) and were given 2 ECTS credits in exchange for their participation. Inclusion criteria were: 18–30 years old, full-time students, German as mother tongue or proficiency. Self-reported mental illness served as an exclusion criterion. Data collection was conducted exclusively online and ended after 202 respondents had been enrolled (M = 21.98 years old, SD = 2.30 years; 133 females).

### Experimental task

Qualtrics Software (Version February 2021, Provo, UT, USA) was used to design the online survey and collect the data. All participants received explanations regarding the alleged nature of the study. The study’s experimental protocols along with the written informed consent had been previously approved by the ethics committee of the Institute of Psychology at the University of Bern (approval code: 2016-02098) and are in accordance with the Declaration of Helsinki. The communicated purpose of the study was the ability to foresee social situations. After signing the informed consent form, the participants could proceed with the online experiment. The experimental task required participants to give likelihood estimates for experiencing the 48 events in a fully randomized fashion and started with a familiarization period, which included three examples. Each trial/scenario was displayed for 10 s and showed a single still animation of a male or female silhouette (whose gender was congruent with the participant’s gender) in the middle upper part of the screen, above a one-sentence description of the target situation. Participants had been instructed before the start of the experiment that they should state their subjective likelihood of occurrence for each situation displayed. Each scenario was presented twice, once in a personal and once in in a general population condition (wherefore each participant saw 96 scenarios). In the personal condition, participants rated the likelihood for themselves experiencing the described scenario in the future. In the general population condition, they provided an average regarding all persons constituting the general population (i.e., a score for the average person of the general population). At the bottom of the screen there was a visual analogue scale (VAS) with which the participants selected a percentage from 0% (left side) to 100% (right side). The VAS always started with the slider on the middle position (50%). Participants had ten seconds to decide: if the time window passed and the slider of the VAS had not been moved, a reminder was shown on the top of the screen that one cannot proceed to the next trial until a decision is made.

### Analysis

#### Statistical design

We used linear mixed modeling as implemented in the GAMLj module in jamovi (The jamovi project (2020). jamovi. (Version 1.6.16) [Computer Software]. Retrieved from https://www.jamovi.org). To answer the first two research questions (H1 and H2), i.e., the valence and gender questions, we ran a linear mixed model with *gender* as between-subjects factor and *target* (self and general population) and *valence* of scenarios (positive and negative) as within-subjects factors. To investigate the effects of sociality and attachment anxiety and avoidance on optimism bias (sociality question; H3-H10), we ran a separate linear mixed model with *gender* as between-subjects factor and the following within-subjects factors: *sociality* (alone and social events) and *valence* of scenarios (positive and negative). *ANX* and *AVO* (both continuous predictors) were between-subjects variables as well. We used the simple effects as implemented in jamovi to investigate individual condition differences for sociality, valence, target, and gender. Significant interactions for attachment were post hoc assessed using the estimated marginal means as implemented in jamovi. For all figures and to elucidate interaction effects where the prior simple effects testing did not elucidate the origin, we aggregated data across scenarios for each participant. Subsequently, gender differences were specified using t tests.

#### Power analysis

We performed 1000 Monte Carlo style simulations using a custom-build script in MATLAB R2017 (MathWorks, Natick, MA) that simulated each trial for each participant prior to running the same analyses as in the current paper. See “Supplementary Materials S1” for further information on this power analysis.

## Supplementary Information

Supplementary Information.

## Data Availability

All data underlying the paper are publicly available at osf.io with the https://doi.org/10.17605/OSF.IO/DVUET.
