# Supplementary material for: Optimism bias and its relation to scenario valence, gender, sociality, and insecure attachment
Source: Sci Rep. 2022 Nov 2;12:18534. doi: 10.1038/s41598-022-22031-4 (PMC9630313; doi:10.1038/s41598-022-22031-4)
Supplement: Supplementary file 1 — Supplementary Information. [file 41598_2022_22031_MOESM1_ESM.docx]

**Supplementary Materials**

**Supplementary Methods**

*Power analysis.* We performed a Monte Carlo style simulation using a custom-build script in Matlab that simulated each trial for each participant, using the random number generator to replace all datapoints prior to running the same analysis as in the paper. We then performed simulations with increasing effect sizes as measured in standardized mean differences that were added to randomly generated likelihood estimates. We did this for each model (1,000 simulations per tested effect size), in order to test what effect size the present sample size would be able to detect for our different hypotheses if several of them were correct at once. The number of randomly generated observations in each simulation was 19 392 for Model 1 (2 targets × 48 scenarios × 202 participants) and 9 696 for model 2 (48 scenarios × 202 participants).

Gender interactions were implemented by adding (or subtracting) effect sizes to the already existing ones within the women sample only. Hypotheses of direction were in line with those from the main manuscript (Table 1). Because the hypotheses tested not only main effects but also interactions, some of these effects could be considered as additive. For example, a main effect of sociality of 0.02 standard deviations, plus an interaction effect of sociality with valence would lead to the positive and social condition being 0.04 standard deviations higher than the average response.

For model two, we additionally had to implement effects for covariates (attachment anxiety and attachment avoidance) and a valence main effect. To realistically mirror the data, attachment anxiety and avoidance were correlated at the same level as in the original data, using Matlab’s copularnd function. The impact of attachment was implemented by taking the participants’ z-score in the respective measures, multiplying it by increasing fractions of standard deviations of the likelihood estimates, and then adding(/subtracting) the results upon the likelihood estimate. Results are given as percentage of simulations that were significant at p<0.05.

**Supplementary Results**

Exact results for models 1 (hypotheses H1 and H2) and 2 (hypotheses H3 to H10) with all factors included are given in tables S1 and S2.

**Table S1**. The first linear mixed model for hypotheses H1-H2

|  | | **F** | | **Num df** | | **Den df** | | **P** | |
| --- | --- | --- | --- | --- | --- | --- | --- | --- | --- |
| Target |  | 294.95 |  | 1 |  | 19138.0 |  | < .001 |  |
| Valence |  | 5.93 |  | 1 |  | 46.1 |  | 0.019 |  |
| Gender |  | 0.07 |  | 1 |  | 200.0 |  | 0.796 |  |
| Target ✻ Valence |  | 147.87 |  | 1 |  | 19138.0 |  | < .001 |  |
| Target ✻ Gender |  | 2.66 |  | 1 |  | 19138.0 |  | 0.103 |  |
| Valence ✻ Gender |  | 7.59 |  | 1 |  | 19138.0 |  | 0.006 |  |
| Target ✻ Valence ✻ Gender |  | 7.03 |  | 1 |  | 19138.0 |  | 0.008 |  |

**Table S2.** The second linear mixed model for hypotheses H3-H10

|  | | **F** | | **Num df** | | **Den df** | | **P** | |
| --- | --- | --- | --- | --- | --- | --- | --- | --- | --- |
| Valence |  | 9.637 |  | 1 |  | 44.2 |  | 0.003 |  |
| Sociality |  | 0.170 |  | 1 |  | 44.2 |  | 0.682 |  |
| Gender |  | 0.002 |  | 1 |  | 196.0 |  | 0.962 |  |
| Attachment avoidance |  | 0.267 |  | 1 |  | 196.0 |  | 0.606 |  |
| Attachment anxiety |  | 2.781 |  | 1 |  | 196.0 |  | 0.097 |  |
| Valence ✻ Sociality |  | 0.017 |  | 1 |  | 44.2 |  | 0.897 |  |
| Valence ✻ Gender |  | 5.144 |  | 1 |  | 9432.0 |  | 0.023 |  |
| Sociality ✻ Gender |  | 7.100 |  | 1 |  | 9432.0 |  | 0.008 |  |
| Sociality ✻ Attachment avoidance |  | 0.526 |  | 1 |  | 9432.0 |  | 0.469 |  |
| Gender ✻ Attachment avoidance |  | 1.496 |  | 1 |  | 196.0 |  | 0.223 |  |
| Valence ✻ Attachment avoidance |  | 12.068 |  | 1 |  | 9432.0 |  | < .001 |  |
| Valence ✻ Attachment anxiety |  | 45.743 |  | 1 |  | 9432.0 |  | < .001 |  |
| Sociality ✻ Attachment anxiety |  | 0.487 |  | 1 |  | 9432.0 |  | 0.485 |  |
| Gender ✻ Attachment anxiety |  | 0.001 |  | 1 |  | 196.0 |  | 0.967 |  |
| Valence ✻ Sociality ✻ Gender |  | 0.403 |  | 1 |  | 9432.0 |  | 0.526 |  |
| Sociality ✻ Gender ✻ Attachment avoidance |  | 0.749 |  | 1 |  | 9432.0 |  | 0.387 |  |
| Valence ✻ Sociality ✻ Attachment avoidance |  | 4.852 |  | 1 |  | 9432.0 |  | 0.028 |  |
| Valence ✻ Gender ✻ Attachment avoidance |  | 7.233 |  | 1 |  | 9432.0 |  | 0.007 |  |
| Sociality ✻ Gender ✻ Attachment anxiety |  | 2.195 |  | 1 |  | 9432.0 |  | 0.138 |  |
| Valence ✻ Sociality ✻ Attachment anxiety |  | 5.110 |  | 1 |  | 9432.0 |  | 0.024 |  |
| Valence ✻ Gender ✻ Attachment anxiety |  | 5.415 |  | 1 |  | 9432.0 |  | 0.020 |  |
| Valence ✻ Sociality ✻ Gender ✻ Attachment avoidance |  | 0.792 |  | 1 |  | 9432.0 |  | 0.373 |  |
| Valence ✻ Sociality ✻ Gender ✻ Attachment anxiety |  | 5.288 |  | 1 |  | 9432.0 |  | 0.021 |  |
|  | | | | | | | | | |

*Power analysis:* The 0-effect models could be seen as a sanity check to indicate whether random results might lead to significant effects on any of the looked-for results. This was not the case, because probabilities in that condition ranged between 3% and 8 % in both models (see Tables S3 and S4).

As can be seen in Table S3, the analysis for model 1 appeared to be sufficiently powered to find the interactions of hypotheses H1 and H2 in more than 85% of cases if the effect size amounted to at least 0.18 standard differences. Table S4 demonstrates that H3-H5 would be significant in more than 84% of cases at effect sizes of 0.18 or more standard deviations of the likelihood estimates.

Only for H6 was a slightly bigger effect size necessary to reach said level: at 0.26 standard deviations, it reached a probability of 82% to show significance.

H7-H10 would reach levels of significance in more than 82% of cases at effect sizes of 0.14 standard deviations of likelihood estimation (as multiplied with the participants’ z-standardized attachment anxiety or avoidance scores).

**Table S3**. Summary of Monte Carlo simulations for model 1.

| Power analysis: percentage of simulations with significant results (p<0.05) for Model 1 | | | | | | | | |
| --- | --- | --- | --- | --- | --- | --- | --- | --- |
|  | Effect size in standard deviation difference for each hypothesis | | | | | | | |
|  | 0.00 | 0.02 | 0.04 | 0.06 | 0.08 | 0.10 | 0.12 | 0.14 |
| Gender | 6.5% | 5.1% | 5.3% | 3.7% | 5.4% | 6.2% | 5.0% | 5.4% |
| Target | 4.1% | 6.9% | 13.5% | 19.9% | 35.3% | 52.4% | 68.5% | 81.8% |
| Valence | 4.3% | 4.7% | 4.4% | 3.9% | 4.4% | 4.3% | 4.5% | 4.7% |
| Gender × Target | 4.5% | 5.1% | 5.3% | 4.0% | 4.1% | 4.2% | 5.0% | 6.2% |
| Gender × Valence | 5.1% | 4.8% | 5.3% | 3.9% | 4.9% | 4.9% | 4.9% | 5.7% |
| Target × Valence | 4.4% | 7.9% | 21.4% | 39.4% | 62.2% | 81.3% | 93.4% | 97.8% |
| Gender × Target × Valence | 4.2% | 6.2% | 11.4% | 17.0% | 29.5% | 41.3% | 50.8% | 62.3% |
|  | Effect size in standard deviation difference for each hypothesis | | | | | | | |
|  | 0.16 | 0.18 | 0.20 | 0.22 | 0.24 | 0.26 | 0.28 | 0.30 |
| Gender | 3.8% | 5.5% | 7.0% | 6.5% | 4.8% | 5.6% | 4.3% | 4.4% |
| Target | 90.2% | 95.0% | 97.7% | 99.4% | 99.6% | 100.0% | 100.0% | 100.0% |
| Valence | 4.1% | 4.2% | 3.6% | 4.9% | 4.0% | 5.3% | 4.2% | 3.6% |
| Gender × Target | 3.8% | 4.9% | 4.2% | 3.9% | 4.4% | 6.2% | 4.4% | 4.0% |
| Gender × Valence | 3.8% | 4.1% | 4.6% | 4.2% | 5.1% | 5.7% | 4.4% | 4.3% |
| Target × Valence | 99.7% | 100.0% | 100.0% | 100.0% | 100.0% | 100.0% | 100.0% | 100.0% |
| Gender × Target × Valence | 75.3% | 86.1% | 91.9% | 96.4% | 98.2% | 98.9% | 99.7% | 100.0% |

*The Supplementary Table S3 reports the percentage of the 1,000 performed simulations that were significant at p <0.05, with estimated likelihood of events occurring being the dependent variable. It gives the percentage for progressively increased effect sizes as standard deviations of estimated likelihood of an event occurring with the present sample of 202 participants. These effect sizes are assigned following Hypotheses H1 and H2 from the main manuscript.*

**Table S4**. Summary of Monte Carlo simulations for model 2.

| Power analysis: percentage of simulations with significant results (p<0.05) for Model 2 | | | | | | | | |
| --- | --- | --- | --- | --- | --- | --- | --- | --- |
|  | Effect size in standard deviation difference for each hypothesis | | | | | | | |
|  | 0.00 | 0.02 | 0.04 | 0.06 | 0.08 | 0.10 | 0.12 | 0.14 |
| Gender | 4.6% | 4.9% | 6.8% | 12.5% | 16.1% | 21.5% | 25.9% | 34.2% |
| Sociality | 4.7% | 11.4% | 35.4% | 72.2% | 90.7% | 98.0% | 99.9% | 100.0% |
| Valence | 4.6% | 6.5% | 8.3% | 13.0% | 22.1% | 28.7% | 37.1% | 48.5% |
| Attachment Anxiety | 6.6% | 9.7% | 19.7% | 30.4% | 44.8% | 59.1% | 71.9% | 82.4% |
| Attachment Avoidance | 5.7% | 9.7% | 18.2% | 30.0% | 45.2% | 58.7% | 72.9% | 83.5% |
| Gender × Sociality | 4.9% | 6.0% | 9.5% | 17.1% | 22.8% | 38.1% | 47.4% | 61.3% |
| Gender × Valence | 4.9% | 4.6% | 6.5% | 7.8% | 10.6% | 12.8% | 15.8% | 21.8% |
| Sociality × Valence | 5.1% | 12.8% | 36.2% | 64.5% | 89.6% | 98.1% | 99.4% | 100.0% |
| Gender × Attachment Avoidance | 5.4% | 3.7% | 4.5% | 4.5% | 4.2% | 4.7% | 4.8% | 5.4% |
| Sociality × Attachment Avoidance | 8.1% | 17.4% | 35.2% | 53.4% | 72.4% | 88.3% | 95.5% | 99.1% |
| Valence × Attachment Avoidance | 6.5% | 8.3% | 12.7% | 19.6% | 24.7% | 36.2% | 43.7% | 54.4% |
| Gender × Attachment Anxiety | 6.2% | 4.6% | 5.0% | 4.3% | 4.4% | 6.0% | 4.4% | 4.3% |
| Sociality × Attachment Anxiety | 8.8% | 18.6% | 34.6% | 53.9% | 72.0% | 84.3% | 94.9% | 98.9% |
| Valence × Attachment Anxiety | 4.8% | 6.9% | 11.6% | 20.7% | 24.1% | 37.5% | 46.9% | 53.5% |
| Gender × Sociality × Valence | 4.6% | 5.8% | 8.1% | 10.9% | 15.6% | 21.2% | 29.0% | 37.7% |
| Gender × Sociality × Attachment Avoidance | 4.6% | 4.1% | 5.0% | 6.2% | 4.2% | 5.5% | 3.8% | 4.2% |
| Gender × Valence × Attachment Avoidance | 5.2% | 3.5% | 3.5% | 5.1% | 5.1% | 3.9% | 4.7% | 4.8% |
| Sociality × Valence × Attachment Avoidance | 7.6% | 10.3% | 19.0% | 34.8% | 47.0% | 63.2% | 73.9% | 83.6% |
| Gender × Sociality × Attachment Anxiety | 4.7% | 5.1% | 4.8% | 5.7% | 3.9% | 4.7% | 5.3% | 6.0% |
| Gender × Valence × Attachment Anxiety | 3.8% | 5.1% | 4.4% | 5.3% | 4.3% | 5.2% | 3.7% | 3.7% |
| Sociality × Valence × Attachment Anxiety | 5.9% | 10.7% | 18.4% | 32.6% | 47.0% | 63.4% | 74.5% | 82.9% |
| Gender × Sociality × Valence × Attachment Avoidance | 4.8% | 4.7% | 5.4% | 5.8% | 4.4% | 4.5% | 6.1% | 4.0% |
| Gender × Sociality × Valence × Attachment Anxiety | 4.6% | 5.1% | 5.2% | 5.2% | 3.8% | 5.4% | 4.4% | 5.3% |
|  | Effect size in standard deviation difference for each hypothesis | | | | | | | |
|  | 0.16 | 0.18 | 0.20 | 0.22 | 0.24 | 0.26 | 0.28 | 0.30 |
| Gender | 45.0% | 54.1% | 63.0% | 70.7% | 78.1% | 86.2% | 88.5% | 93.6% |
| Sociality | 100.0% | 100.0% | 100.0% | 100.0% | 100.0% | 100.0% | 100.0% | 100.0% |
| Valence | 60.1% | 71.1% | 82.2% | 87.1% | 92.2% | 94.2% | 97.6% | 99.1% |
| Attachment Anxiety | 90.0% | 94.7% | 97.9% | 99.2% | 99.8% | 99.9% | 100.0% | 100.0% |
| Attachment Avoidance | 89.0% | 95.1% | 98.4% | 99.0% | 99.7% | 99.9% | 100.0% | 100.0% |
| Gender × Sociality | 74.0% | 84.3% | 90.4% | 95.0% | 96.2% | 99.1% | 99.7% | 99.6% |
| Gender × Valence | 27.2% | 30.0% | 35.9% | 45.3% | 50.0% | 56.8% | 63.9% | 69.7% |
| Sociality × Valence | 100.0% | 100.0% | 100.0% | 100.0% | 100.0% | 100.0% | 100.0% | 100.0% |
| Gender × Attachment Avoidance | 5.3% | 4.7% | 3.8% | 5.6% | 6.0% | 4.8% | 5.2% | 3.8% |
| Sociality × Attachment Avoidance | 99.6% | 99.8% | 100.0% | 100.0% | 100.0% | 100.0% | 100.0% | 100.0% |
| Valence × Attachment Avoidance | 64.0% | 72.8% | 83.2% | 85.2% | 92.9% | 95.8% | 97.5% | 98.3% |
| Gender × Attachment Anxiety | 5.0% | 5.7% | 4.2% | 5.7% | 6.3% | 4.7% | 4.6% | 4.1% |
| Sociality × Attachment Anxiety | 99.6% | 99.9% | 99.9% | 100.0% | 100.0% | 100.0% | 100.0% | 100.0% |
| Valence × Attachment Anxiety | 66.5% | 70.3% | 83.3% | 87.4% | 92.8% | 95.6% | 97.0% | 98.9% |
| Gender × Sociality × Valence | 44.1% | 53.8% | 63.9% | 72.3% | 78.6% | 82.0% | 91.6% | 94.0% |
| Gender × Sociality × Attachment Avoidance | 4.3% | 4.7% | 4.4% | 5.6% | 5.5% | 6.8% | 6.0% | 4.2% |
| Gender × Valence × Attachment Avoidance | 5.2% | 4.6% | 5.2% | 5.5% | 5.1% | 6.5% | 6.4% | 5.2% |
| Sociality × Valence × Attachment Avoidance | 91.2% | 94.7% | 97.5% | 99.3% | 99.4% | 99.8% | 100.0% | 100.0% |
| Gender × Sociality × Attachment Anxiety | 4.9% | 6.4% | 4.2% | 4.5% | 6.4% | 6.3% | 4.6% | 4.2% |
| Gender × Valence × Attachment Anxiety | 5.6% | 5.2% | 6.0% | 6.2% | 5.9% | 5.5% | 5.5% | 6.1% |
| Sociality × Valence × Attachment Anxiety | 91.3% | 95.4% | 97.9% | 99.0% | 99.9% | 99.9% | 100.0% | 99.9% |
| Gender × Sociality × Valence × Attachment Avoidance | 4.7% | 5.0% | 3.7% | 5.9% | 4.9% | 5.7% | 4.6% | 3.8% |
| Gender × Sociality × Valence × Attachment Anxiety | 5.1% | 5.2% | 3.4% | 4.9% | 7.1% | 5.1% | 5.9% | 3.3% |

*The Supplementary Table S4 reports the percentage of the 1,000 performed simulations that were significant at p <0.05, with estimated likelihood of events occurring being the dependent variable. It gives the percentage for progressively increased effect sizes as standard deviations of estimated likelihood of an event occurring with the present sample of 202 participants. These effect sizes are assigned following Hypotheses H3 to H10 from the main manuscript. For H7 to H10 they were applied by multiplying the participants’ z-standardized attachment anxiety/attachment avoidance scores by the assigned fraction of standard deviation of the likelihood estimate and adding or subtracting these from the randomly generated levels, as indicated by these hypotheses.*

**Supplementary Discussion**

*Power analysis.* The Monte Carlo analysis of both models indicates that our study - using mixed models as we did - was adequately powered to detect medium to small effect sizes of often less than 0.2 standard deviations, in some cases even just 0.1 standard deviations difference. It is important to note that this was the case although effects can interact with each other. The model by and large still managed to find and separate these effects from each other. Furthermore, the model overestimates null effect, as seen from the sanity checks performed assuming the existence of no effects at all.

**Supplementary Figures**


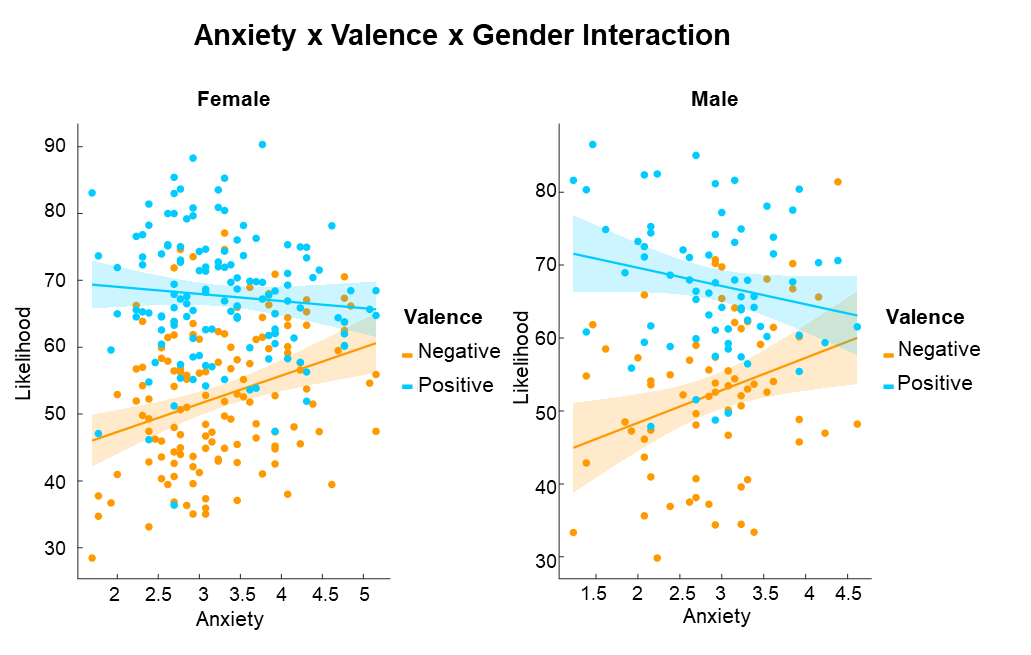


Supplementary Figure S1. Scatter plot displaying the interaction between attachment anxiety, valence, and gender.

## Table S5. List of events included in the study

| **Scenario** | **Perceived valence** | **Perceived sociality** | **Mean Valence (SD)** | **Mean Sociality (SD)** |
| --- | --- | --- | --- | --- |
| Paying bills. | Negative | Low | -12.0 (19.7) | 43.6 (36.0) |
| Being lonely in old age. | Negative | Low | -39.2 (12.7) | 36.8 (32.3) |
| Carrying the trash outside. | Negative | Low | -1.2 (16.2) | 40.4 (37.2) |
| Finding rotten food in the refrigerator. | Negative | Low | -28.7 (15.6) | 39.0 (31.0) |
| Not having access to a power socket when traveling. | Negative | Low | -15.5 (16.3) | 41.6 (33.1) |
| Getting food poisoning. | Negative | Low | -40.1 (11.8) | 30.8 (30.4) |
| Experiencing a huge financial loss. | Negative | Low | -39.2 (13.5) | 51.1 (29.2) |
| Finding food past its expiration date in the cupboard. | Negative | Low | -21.2 (14.8) | 42.6 (31.1) |
| Being bitten by a dog. | Negative | Low | -38.6 (14.0) | 48.0 (35.0) |
| Being alone on one's birthday. | Negative | Low | -34.2 (16.0) | 36.5 (33.5) |
| Getting a sunburn. | Negative | Low | -28.8 (16.1) | 21.3 (28.9) |
| Getting stuck in an elevator. | Negative | Low | -32.3 (15.1) | 47.0 (33.0) |
| Saying something that upsets a friend. | Negative | High | -27.4 (13.9) | 80 (21) |
| A neighbor listening to music too loudly when one wants to sleep. | Negative | High | -28.0 (14.5) | 75.8 (25) |
| Being lied to by one's partner. | Negative | High | -39.7 (14.7) | 74.9 (27.8) |
| Having to work with someone one doesn't like. | Negative | High | -27.6 (13.4) | 75.5 (23.2) |
| Waiting fifteen minutes for a friend who is late. | Negative | High | -18.4 (12.0) | 74.0 (25.8) |
| The airplane one is waiting to board is ten minutes late. | Negative | High | -16.1 (13.6) | 79.3 (24.2) |
| Being misunderstood when trying to explain one's point of view. | Negative | High | -23.7 (15.5) | 75.4 (21.8) |
| Being responsible for someone's emotional suffering. | Negative | High | -38 (14.7) | 78.9 (24.8) |
| Being mistaken for another person by someone. | Negative | High | -4.1 (11.0) | 70.1 (26.0) |
| Arguing with a relative | Negative | High | -31.2 (12.9) | 80.8 (20.1) |
| Someone is making fun of you. | Negative | High | -33.1 (17.2) | 66.2 (30.5) |
| Accidentally pouring a drink over a friend. | Negative | High | -20.9 (14.4) | 75.8 (25.9) |
| Getting five francs interest from the bank at the end of the month. | Positive | Low | 16.1 (18.1) | 29.4 (26.9) |
| Getting a haircut. | Positive | Low | 19.9 (16.2) | 40.4 (35.2) |
| Being able to pay in a store with a credit card | Positive | Low | 10.7 (15.9) | 37.8 (33.9) |
| Living the lifestyle one has always wanted. | Positive | Low | 36.6 (18.5) | 44.5 (32.1) |
| Seeing a shooting star. | Positive | Low | 30.2 (15.6) | 27.5 (30.3) |
| Learning a new language. | Positive | Low | 29.0 (16.3) | 28.3 (28.5) |
| Win an all-inclusive trip for one person. | Positive | Low | 34.7 (16.4) | 33.8 (34.2) |
| Make a purchase of more than 50 francs for one's own pleasure. | Positive | Low | 17.5 (16.2) | 27.5 (27.6) |
| Living a healthy and active life until death. | Positive | Low | 36.2 (16.0) | 37.2 (31.9) |
| Finding a free seat on the bus. | Positive | Low | 17.5 (18.7) | 61.0 (32.2) |
| Seeing an interesting offer in the supermarket. | Positive | Low | 17.5 (14.6) | 37.9 (33.3) |
| Enjoying a quiet afternoon alone. | Positive | Low | 29.5 (15.8) | 23.3 (27.9) |
| Meeting a colleague at the cinema. | Positive | High | 21 (15.6) | 76.1 (22.2) |
| Being asked for directions by someone on the street. | Positive | High | 9.2 (13.7) | 75.3 (24.5) |
| Saying hello to a neighbor in the stairwell. | Positive | High | 21.8 (16.9) | 80.2 (21.6) |
| Having relatives greet you in a friendly way at a family gathering. | Positive | High | 32.3 (16.4) | 80.1 (21.3) |
| Cooking vegetarian food for friends. | Positive | High | 25.9 (19.2) | 78.0 (24.7) |
| Meeting an old friend by chance on the street. | Positive | High | 21.6 (16.3) | 76.2 (21.6) |
| A new neighbor comes over to introduce themselves. | Positive | High | 20.4 (16.9) | 76.0 (24.3) |
| Going to a party with friends and having fun. | Positive | High | 35.4 (14.0) | 81.8 (20.7) |
| Seeing two people kissing on the street. | Positive | High | 11.1 (17.5) | 75.8 (27.5) |
| Going on vacation with one's partner. | Positive | High | 36.0 (14.5) | 82.8 (18.8) |
| Donating money to a person in need. | Positive | High | 29.2 (16.8) | 68.1 (31.6) |
| Getting a birthday gift from friends. | Positive | High | 34.6 (15.7) | 70.0 (29.1) |
